# The influence of the direction of J-tip on the placement of a subclavian catheter: real time ultrasound-guided cannulation versus landmark method, a randomized controlled trial

**DOI:** 10.1186/1471-2253-14-11

**Published:** 2014-02-28

**Authors:** Ah-Young Oh, Young-Tae Jeon, Eun-Joo Choi, Jung-Hee Ryu, Jung-Won Hwang, Hee-Pyoung Park, Sang-Hwan Do

**Affiliations:** 1 Department of Anesthesiology and Pain Medicine, Seoul National University Bundang Hospital, Seongnam, Korea; 2 Department of Anesthesiology and Pain Medicine, Seoul National University College of Medicine, Seoul, Korea; 3 Department of Anesthesiology and Pain Medicine, Seoul National University Hospital, Seoul, Korea

**Keywords:** Central venous catheterization, Subclavian vein, Ultrasound

## Abstract

**Background:**

It has been reported that the direction of the guidewire J-tip is associated with misplacement of a central venous catheter. We hypothesized that real-time ultrasound-guided infraclavicular subclavian venous cannulation would be less influenced by the direction of guidewire J-tip compared to landmark method.

**Methods:**

Sixty adult patients who required subclavian venous catheterization for neurosurgery were enrolled in this prospective randomized controlled study. Patients were randomly divided into a landmark group (n = 30) or an ultrasound group (n = 30). After the subclavian vein was punctured, the guidewire was advanced with the guidewire J-tip directed cephalad. Misplacement or advancement failure of the guidewire was regarded as an unsuccessful placement. Postoperative chest radiography was performed to confirm pneumothorax and the location of the catheter tip.

**Results:**

The two groups were comparable with respect to age, gender, height, and weight distribution. The incidence of unsuccessful guidewire placement was lower in the ultrasound group than in the landmark group (13% vs. 47%, P = 0.01). Among the unsuccessful guidewire placements, the incidence of misplacement were comparable between the groups and were all located in the ipsilateral internal jugular vein (7% vs. 7%). However, the incidence of advancement failure was significantly higher in landmark group (40% vs. 7%, P = 0.005). There were no complications such as pneumothorax or hemothorax.

**Conclusions:**

The proper placement of guidewire was less influenced by the direction of the guidewire J-tip with ultrasound-guided subclavian venous cannulation than with the landmark approach.

## Background

Aberrant placement of a subclavian venous catheter tip may result in incorrect central venous pressure readings or serious complications such as vascular erosion and thrombosis. Various techniques have been suggested to avoid aberrant location of a catheter [1-5]. The direction of the guidewire J-tip is associated with misplacement of a central venous catheter and higher rate of misplacement is reported when guidewire J-tip is directed cephalad [4,5]. Although initial direction of J-tip is important on the determination of ultimate location, rotation could occur inside veins. It explains failure of the guidewire to enter into the superior vena cava even when the J-tip was directed caudad [4].

Compared with the landmark approach, real-time ultrasound-guided cannulation results in a higher success rate, requires fewer attempts, and has a lower rate of mechanical complications; thus, it increases the safety of central venous access via the subclavian route [6].

With ultrasound-guided approaches, the course of needle approaching the vein and the tip of needle properly located in the subclavian vein (SCV) can be visualized, which allows more distance from the tip of needle to vessel wall of opposite site. The usual point of skin puncture is more lateral than that of landmark method and the point entering the lumen of the vein is either at the axillary vein or at the point where it continues as the SCV [6], which allows longer path for guidewire before entering superior vena cava.

We hypothesized that in ultrasound method, the proper positioning of the guidewire would be less influenced by the direction of a guidewire J-tip compared to landmark one during SCV cannulation. We evaluated the success rate of proper guidewire placement while the guidewire J-tip directed cephalad before insertion into a needle hub in ultrasound group and landmark group.

## Methods

With approval of the Institutional Review Board of Seoul National University Bundang Hospital (IRB Number E-1006/050-003), informed consent was obtained from all patients. The study protocol was registered with the Korean Clinical Trials Registry (Number KCT0000083).

In this prospective randomized controlled study, patients between the age of 18 and 75 years (ASA physical status I–III), who required subclavian venous catheterization for neurosurgery at Seoul National University Bundang Hospital throughout October 2010 to November 2011 were included. Patients with chest deformities or significant coagulopathy were excluded. Patients were allocated to two groups, the landmark or ultrasound group, by using a block randomization technique. Randomization was determined with random number tables, and the assignments were concealed in sealed envelopes until immediately before induction of anesthesia.

After the induction of general anesthesia, right subclavian venous catheterization was performed using a double-lumen central venous catheter (Arrow International Inc., Reading, PA, USA) under aseptic conditions. Patients were placed in the supine position, with the head and shoulder in the neutral position. In the landmark group, the skin was punctured at 2–3 cm below the right clavicle, along the mid-clavicular line, toward the upper border of the suprasternal notch; the puncture needle was advanced until the right SCV was punctured. In the ultrasound group, the real-time ultrasound-guided technique was performed using a SonoSite S-nerve (SonoSite, Bothell, WA, USA) equipped with a high-resolution 7.5 mHz transducer. The transducer was covered with a sterile sheath, and ultrasonic gel was used. A colour Doppler technique was used to confirm the vein. The right SCV to be catheterized was located in the longitudinal plane, and the needle was directed at the vein in real time. In both groups, when the free flow of non-pulsatile venous blood appeared, the guidewire was advanced with the J-tip directed cephalad. The ipsilateral IJV and contralateral SCV as well as ipsilateral SCV were scanned to confirm the proper position of guidewire. If the guidewire could not be advanced due to resistance or was seen in the IJV under ultrasound examination, it was regarded as an unsuccessful placement, which was the primary outcome of this study. Patients in whom the SCV catheterization failed or arterial puncture occurred were excluded from the analysis.

Postoperative chest radiography was performed on all patients to visualize a pneumothorax or hemothorax, which was the secondary outcome measures, and the location of the catheter tip.

### Statistical analysis

The sample size calculation was based on the results of a previous study, which reported the incidence of catheter tip malpositioning during infraclavicular subclavian venous cannulation to be 43% when the J-tip was directed cephalad [4]. A sample size of 33 patients per group was required to maintain the incidence of unsuccessful location below 10%, with a power of 80% and an α of 0.05, considering 10% dropouts. The Mann–Whitney *U* test was used to compare mean values between the two groups, and a *χ*^2^ test was performed to compare the gender difference and the incidence of failure of proper guidewire placement. A value of *P* < 0.05 indicated statistical significance.

## Results

**Figure 1:**
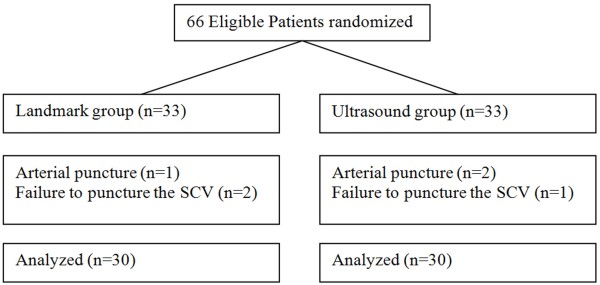
**CONSORT flowchart.** SCV = subclavian vein.

Of the 66 patients enrolled for randomization, six patients were excluded from the study (Figure 1). Three patients had arterial puncture, and in three, the SCV could not be punctured. Both groups were comparable with respect to age, gender, height, and weight distribution (Table 1).

**Table 1:** Patient characteristics

	**Landmark group**	**Ultrasound group**
	**(n = 30)**	**(n = 30)**
Male: female	15:15	16:14
Age, years	50 (16)	51 (14)
Height, cm	163 (8)	163 (8)
Weight, kg	62 (12)	65 (11)

Data are expressed as mean (SD) or number of patients.

The incidence of unsuccessful placement of catheter tips was lower in the ultrasound group than in the landmark group (13% vs. 47%, *P* = 0.01). Of the unsuccessful guidewire placement, the incidence of advancement failure was significantly higher in landmark group (landmark: 40%, 12/30 vs. ultrasound: 7%, 2/30, P = 0.005). The incidence of misplacement among the rest of the patients were comparable between the groups and were all located in the ipsilateral internal jugular vein (landmark: 11%, 2/18 vs. ultrasound: 7%, 2/28) (Table 2). Unsuccessful placements were all corrected by repositioning under ultrasound guidance. There were no adverse effects such as pneumothorax or hemothorax.

**Table 2:** Unsuccessful placement of guidewire

	**Landmark group**	**Ultrasound group**
	**(n = 30)**	**(n = 30)**
Unsuccessful placement	14 (47%)	4 (13%)*
Failure to thread the guidewire	12 (40%)	2 (7%)*
Ipsilateral IJV	2 (7%)	2 (7%)
Complication	0	0

*IJV* internal jugular vein.

*Statistically significant (*P* < 0.05) difference compared to landmark group.

## Discussion

Our study showed that the incidence of unsuccessful guidewire placement was significantly lower during subclavian venous cannulation when ultrasound is used compared to landmark method even when the guidewire J-tip directed cephalad before insertion into a needle hub.

We suggest several explanations for lower incidence of unsuccessful guidewire placement in the ultrasound group. First, the different angle of the venipuncture between the two techniques might be related. With the ultrasound-guided technique, the direction of needle pointed along the long axis of the SCV, facilitating proper guidewire advancement. In contrast, with the landmark technique, the angle of the needle was much flatter against the skin, and the needle pointed toward the suprasternal notch, which can lead to the advance of the guidewire into the IJV. Second, the point of vascular puncture is usually at axillary vein or at the junction where it continues as SCV and is more lateral compared to that of landmark method, which allows the longer path for guidewire before entering superior vena cava. Third, more accurate placement of a needle tip in the vessel is possible and the distance from the tip of needle to the vessel wall of opposite side might be longer because the course of needle passage and the tip of needle can be visualized in real time.

It is recommended that the direction of the guidewire J-tip should be adjusted caudad before fitting into the subclavian puncture needle hub [4]. However, when the J-tip is exposed out of the sheath or its protective cap, it can point in any direction. Sometimes, it is difficult to insert the J-tip and guidewire in the initial direction, depending on the sheath plane [7]. Moreover, rotation of the J-tip can occur inside the vein and the caudad direction of the guidewire J-tip does not allow 100% entry of the guidewire into the SVC. In an experimental design with rigid tubing, the J-tip of the guidewire followed its natural curve into the respective side of the simulated vessel [4]. However, the J-tip may rotate inside veins that are more compliant. We suggest, from the results of this study, that much of these problems related to the direction of guidewire tip could be solved with the use of the real-time ultrasound method.

The head or shoulder position appears to be important for the central venous catheter direction during the infraclavicular subclavian approach [8-10]. Turning the head away from the needle puncture site increases the angle of the SCV and IJV, and causes malpositioning in the IJV [8,10]. Lowering the shoulder position reduces the angle between the SCV and innominate vein, and increases the incidence of catheter misplacement into the ipsilateral IJV [9,10]. In the present study, subclavian venous cannulation was performed with the head and shoulders in a neutral position to exclude any positional effect.

In this study, the incidence of failure to thread the guidewire was high. Failure to thread the guidewire can occur when the J-tip is directed cephalad [5]. If the guidewire met any stiff resistance while advancing into the SCV, we did not advance the guidewire. If we had advanced the guidewire despite the resistance we could have advanced into the IJV more frequently.

The use of ultrasound for subclavian venous cannulation has been controversial for anatomical reasons [11,12]. It has been suggested that the anatomical relationship between the SCV and clavicle makes ultrasound-guided catheterization more difficult and less reliable than a landmark-based insertion technique [13]. However, these previous studies used Doppler techniques, and not a real-time ultrasound-guided technique. A higher success rate and lower incidence of mechanical complications have been well established for real-time ultrasound-guided technique compared with the landmark one [6,14].

There were some limitations to our study. First, this investigation was not-blinded because the catheterization could not be disguised; therefore, there was a possibility of a bias. Second, the ultrasound-guided technique failed in three patients. The real-time ultrasound technique has been rated as technically difficult by participating physicians [6]. As previously suggested, the benefits of the ultrasound technique may not be realized until after a significant learning period [15]. Third, we initially inserted J-tip in the cephalad direction to maximize the chance of the guidewire going up into the IJV, although this is not a good clinical practice when using landmark technique. To compensate for this, the location of guidewire was checked under ultrasound examination in all patients. The last, we could not exclude the possibility of the advantage of lateral approach. It is plausible that the advantages in the ultrasound group with respect to proper guidewire insertion can not only be seen as a consequence of ultrasound usage as well as but also as a consequence of the insertion site.

## Conclusions

We demonstrated that the rate of unsuccessful guidewire placement was significantly lower in real-time ultrasound guided SCV cannulation compared to landmark one even though the guidewire tip was directed cephalad before insertion into a needle hub. We suggest that the guidewire placement is less influenced by the direction of guidewire J-tip in ultrasound method compared to landmark method. From this result, together with the fact that the guidewire placement could be monitored in real time, we concluded that a considerable portion of problems related to the direction of guidewire tip could be resolved with ultrasound method. This could be another benefit of ultrasound-guided SCV cannulation, making the procedure easier, which could be added to already known benefits.

## Competing interests

The authors declare that they have no competing interests.

## Authors’ contributions

AYO contributed to conception and design of the study, wrote the manuscript. YTJ contributed to conception and design of the study, revised the manuscript. HPP contributed to conception and design of the study. EJC, JHR, and JWH contributed to acquisition, analysis, and interpretation of data. SHD revised the manuscript. All authors read and approved the final manuscript.

## Pre-publication history

The pre-publication history for this paper can be accessed here:

http://www.biomedcentral.com/1471-2253/14/11/prepub
